# CTC-Derived Models: A Window into the Seeding Capacity of Circulating Tumor Cells (CTCs)

**DOI:** 10.3390/cells8101145

**Published:** 2019-09-25

**Authors:** Tala Tayoun, Vincent Faugeroux, Marianne Oulhen, Agathe Aberlenc, Patrycja Pawlikowska, Françoise Farace

**Affiliations:** 1 “Circulating Tumor Cells” Translational Platform, CNRS UMS3655 – INSERM US23AMMICA, Gustave Roussy, Université Paris-Saclay, F-94805 Villejuif, France; tala.tayoun@gustaveroussy.fr (T.T.); vincent.faugeroux@laposte.net (V.F.); marianne.oulhen@gustaveroussy.fr (M.O.); agathe.aberlenc@gustaveroussy.fr (A.A.); 2 INSERM, U981 “Identification of Molecular Predictors and new Targets for Cancer Treatment”, F-94805 Villejuif, France; patrycjamarta.pawlikowska@gustaveroussy.fr; 3 Faculty of Medicine, Université Paris Sud, Université Paris-Saclay, F-94270 Le Kremlin-Bicetre, France

**Keywords:** metastasis, tumor-initiating cells (TICs), circulating tumor cells (CTCs), CTC-derived xenografts, CTC-derived ex vivo models

## Abstract

Metastasis is the main cause of cancer-related death owing to the blood-borne dissemination of circulating tumor cells (CTCs) early in the process. A rare fraction of CTCs harboring a stem cell profile and tumor initiation capacities is thought to possess the clonogenic potential to seed new lesions. The highest plasticity has been generally attributed to CTCs with a partial epithelial-to-mesenchymal transition (EMT) phenotype, demonstrating a large heterogeneity among these cells. Therefore, detection and functional characterization of these subclones may offer insight into mechanisms underlying CTC tumorigenicity and inform on the complex biology behind metastatic spread. Although an in-depth mechanistic investigation is limited by the extremely low CTC count in circulation, significant progress has been made over the past few years to establish relevant systems from patient CTCs. CTC-derived xenograft (CDX) models and CTC-derived ex vivo cultures have emerged as tractable systems to explore tumor-initiating cells (TICs) and uncover new therapeutic targets. Here, we introduce basic knowledge of CTC biology, including CTC clusters and evidence for EMT/cancer stem cell (CSC) hybrid phenotypes. We report and evaluate the CTC-derived models generated to date in different types of cancer and shed a light on challenges and key findings associated with these novel assays.

## 1. Introduction

Metastatic spread and its resistance to treatment remain the leading cause of death in cancer patients. This process is fueled by malignant cells that dissociate from the primary tumor and travel through the bloodstream to colonize distant organs. These cells are referred to as “circulating tumor cells” (CTCs) and are able to enter vasculature during the early course of disease. Nonetheless, the majority of the tumor cell population dies during transit as a result of biological and physical constraints such as shear stress and immune surveillance, and only a minor subset of the surviving CTCs (0.01%) acquires the capacity of tumor-initiating cells (TICs) [1,2,3,4]. The outcome of tumor dissemination is dependent on a selection process that favors the survival of a small proportion of cancer cells holding the self-renewal ability of stem cells along with TIC properties, which enables them to seed tumors and reconstitute tumor heterogeneity [5,6,7]. These cells are termed “cancer stem cells” (CSCs), and CTCs holding a CSC phenotype have been detected and associated with high invasiveness and tumorigenicity in many cancers including breast cancer (BC), colorectal cancer, and glioma [8,9,10,11].

An important aspect of CTC research is to study the mechanistic basis behind their TIC properties and explore new CTC-based biomarkers and targeting strategies. The generation of CTC-derived xenografts (CDXs) or CTC-derived cell lines at relevant time points during disease progression is therefore crucial to achieve a longitudinal and functional characterization of these cells, along with in vivo and in vitro pharmacological testing. Although this task remains challenging owing to CTCs scarcity in peripheral blood and technical hurdles related to their enrichment strategies, significant efforts have been made in the establishment of clinically relevant systems to study CTC biology in different cancer types. In this review, we briefly cover basic knowledge of TIC-related properties in CTCs and evaluate the existing CTC-derived models, including both in vivo CDXs and in vitro functional culture assays in different cancers. We also highlight the important findings which have helped unveil new insights into CTC biology and novel therapeutic strategies.

## 2. Brief Glimpse into TIC-Related Properties of CTCs

CTC profile evolves as the initial events of the metastatic cascade take place. Indeed, CTCs undergo reversible phenotypic alterations to achieve intravasation, survival in vasculature and extravasation, known as epithelial-to-mesenchymal transition (EMT). During EMT—a key phenomenon in embryonic development—cancer cells undergo cytoskeletal changes and typically lose their cell–cell adhesion proteins as well as their polarity to become motile cells and intravasate [12,13]. EMT signatures were detectable in CTCs of BC patients [14,15,16,17]. Increasing experimental evidence draws a potential link between EMT and acquisition of stemness [12,13,18,19]. In fact, several EMT-inducing transcription factors have been shown to confer malignancy in neoplastic cells, leading to the emergence of highly aggressive clones with combined EMT/CSC traits [20,21,22,23]. Nevertheless, this association is not universal. Indeed, it has been suggested that the loss of the EMT-inducing factor *Prxx1* is required for cancer cells to colonize organs in vivo, which revert to the epithelial state and acquire CSC traits, thus uncoupling EMT and stemness [19,24,25]. Moreover, the requirement of EMT for CTC dissemination has long been subject to debate. Several studies have shown that mesenchymal features in tumor cells may indeed be dispensable for their migratory activity but could contribute molecularly and phenotypically to chemoresistance [26,27,28]. It is currently hypothesized that CTC subclones displaying an intermediate phenotype between epithelial and mesenchymal have the highest plasticity to adapt to the microenvironment and generate a more aggressive CTC population resistant to conventional chemotherapy and capable of metastatic outgrowth. Our group showed the existence of a hybrid epithelial/mesenchymal (E/M) phenotype in CTCs from patients with non-small cell lung cancer (NSCLC) [29]. Heterogeneous expression of EMT markers within SCLC and NSCLC patient cohorts was described by Hou et al., while Hofman et al. reported the presence of proportions of NSCLC CTCs which expressed the mesenchymal marker vimentin and correlated with shorter disease-free survival [30,31]. Recent data in metastatic BC patients showed the enrichment of CTC subpopulations with a CSC^+^/partial EMT^+^ signature in patients post-treatment, which correlated with worse clinical outcome [32]. Indeed, the CTC population is described as a highly heterogeneous pool of tumor cells with low numbers of metastasis-initiating cells (MICs) that are sometimes prone to apoptosis [33]. The different factors influencing MIC properties of CTCs and their survival underlie the complexity and inefficiency of organ invasion and macro-metastases formation, relevant both clinically and in experimental mouse models [4,34,35]. Recent advances in single-cell technologies have unraveled CTC-specific genetic mutations and profiling of the CTC population thus points out the emergence of subclones with dynamic phenotypes that contribute to the evolution of the tumor genome during disease progression and treatment [36,37,38,39]. CTCs are less frequently found in clusters, also termed “circulating tumor microemboli” (CTM), which travel as 2–50 cells in vasculature and present extremely enhanced metastatic competency [40]. This can be explained by the survival advantage they hold over single CTCs, as CTM were shown to escape anoikis as well as stresses in circulation [30,41]. A recent report showed that these characteristics are due to CSC properties of CTM, notably a CD44-directed cell aggregation mechanism that forms these clusters, promotes their survival and favors polyclonal metastasis [42]. Another group also investigated the factors behind CTM metastatic potential: Gkountela et al. reported that CTC clusters from BC patients and CTC cell lines exhibit a DNA methylation pattern distinct from that of single CTCs and which represents targetable vulnerabilities [43]. Moreover, CTC-neutrophils clusters are occasionally formed in the bloodstream and in vivo evidence shows that this association triggers cell cycle progression and thus drives metastasis formation in BC [44].

## 3. Brief Introduction to CTC Enrichment and Detection Strategies

A plethora of technologies have been developed over the last decade to respond to specific CTC applications. CTC identification remains a technically challenging task due to the extreme phenotypic heterogeneity and rarity of these cells in the bloodstream and therefore requires methods with high sensitivity and specificity. Enrichment strategies can be based on either biological properties (i.e., cell-surface markers) or physical characteristics (i.e., size, density, electric charge) and are usually combined with detection techniques (e.g., immunofluorescence, immunohistochemistry, FISH) to identify CTCs. CTC capture relies on a positive selection among normal blood cells or a negative selection by leukocyte depletion. Among biologically-based technologies is the CellSearch system (Menarini-Silicon Biosystem, Bologna, Italy). It is the most commonly applied assay for CTC enumeration in which CTCs are captured in whole blood by EpCAM (epithelial cell adhesion molecule)-coated immunomagnetic beads followed by fluorescent detection using anti-cytokeratins (CK 8, CK 18, CK 19), anti-CD45 (leukocyte marker), and a nuclear stain (DAPI). It is the only technology cleared by the US Food and Drug Administration to aid in prognosis for patients with metastatic breast, prostate, and colorectal cancer [45,46,47,48,49]. Although standardized and reproducible, this method has a limited sensitivity most likely due to failure in recognizing cells undergoing EMT and thus inevitably misses an aggressive and clinically relevant CTC subpopulation. Platforms relying on the depletion of leukocytes (negative selection) are being investigated and used to overcome this bias. One example is the widely used RosetteSep technique which enriches CTCs without phenotypic a priori by excluding CD45^+^ and CD36^+^ cells in rosettes and eliminating them in a Ficoll-Paque PLUS density-gradient centrifugation. Physical property-based methods including filtration systems have been developed to capture CTCs based on their large size compared to leukocytes, notably the ISET^®^ (*Isolation by Size of Tumor Cells*) (RareCells Diagnostics, Paris, France) and the ScreenCell^®^ (Paris, France) methods, which are able to detect CTCs as well as CTM using microporous polycarbonate filters [50,51]. In line with this notion, we and others have reported an overall higher recovery rate using ISET compared to CellSearch for CTC enumeration in NSCLC and prostate cancer patients [31,52]. Our lab developed a novel CTC detection approach combining ISET filtration with a FISH assay, optimized for the detection of *ALK*- or *ROS1*-rearranged pattern of NSCLC CTCs on filters [53,54]. To ensure a wider coverage of CTC heterogeneity, new devices are being developed (and some commercially) such as the CTC-iChip which relies on both biological and physical properties of CTCs: it applies size-based filtration using microfluidics processing, followed by positive selection of CTCs with EpCAM-conjugated beads or negative selection with CD45^−^-coated beads to deplete hematopoietic cells [55]. Different technologies have been implemented to isolate live CTCs (without a fixation step) and perform subsequent functional studies. Some strategies have integrated isolation protocols for molecular analysis of single CTCs. One example is the DEPArray^TM^ (Silicon Biosystems S.p.A., Bologna, Italy), a microfluidic system which sorts live single CTCs based on image selection followed by entrapment of CTCs inside dielectrophoretic cages [56,57,58]. FACS has also been adapted for molecular characterization of CTCs as well as their isolation in the aim of xenograft establishment [59].

At this point, none of the technologies fully respond to the phenotypic heterogeneity of CTCs. Indeed, each method has its own advantages and limitations and researchers have based the development of capture strategies on the specific aim of further CTC characterization studies. New insights in CTC biology should be integrated into current enrichment, detection, and isolation techniques to optimize the process and improve their reliability. As shown in Table 1, RosetteSep and FACS have been used for CDX establishment. Enrichment using RosetteSep may be advantageous owing to the lack of phenotypic a priori on tumorigenic CTCs and a higher recovery rate.

## 4. CTC-Derived Xenografts

Patient-derived xenograft (PDX) technology has rapidly emerged as a standard translational research platform to improve understanding of cancer biology and test novel therapeutic strategies [60]. PDXs are generated by implantation of surgically-removed tumor tissue (primary or metastasis) into immunodeficient mice. Although these models have proven utility as a preclinical tool in many cancers, their feasibility remains challenged by limited tumor tissue availability, as single-site biopsies may be impossible or detrimental in some malignancies [61]. This limitation can be overcome by the generation of CDX models after enrichment of CTCs collected from a readily accessible blood draw and subsequent injection into immunodeficient mice [62,63,64]. Nevertheless, it is noteworthy that CDX development still presents an enormous challenge due to low CTC prevalence in several cancers. Until now, CDXs have been established in breast, melanoma, lung and prostate cancer and are discussed in this section (Table 1).

In 2013, Baccelli et al. reported the first experimental proof that primary human luminal BC CTC populations contain MICs in a xenograft assay. Injection of CTCs from 110 patients was performed. Six recipient mice developed bone, lung, and liver metastases within 6–12 months after CTC transplant (~1000 CTCs) from three patients with advanced metastatic BC. Cell sorting analysis of the MIC-containing population shared a common EpCAM^+^CD44^+^MET^+^CD47^+^ phenotype, highlighting a CSC characteristic of CTCs. The authors also showed that the number of CTCs positive for these markers strongly correlated with decreased progression-free survival of metastatic BC patients. This study has therefore revealed a first phenotypic identification of luminal BC CTCs with MIC properties, making them an attractive tool to track and potentially target metastatic development in BC [59]. A second group derived a CDX model from a metastatic triple-negative BC (TNBC) patient for the first time. The patient selected for CDX establishment had advanced TNBC with a very high CTC count obtained with CellSearch analysis (969 CTCs and 74 CTC clusters/7.5 mL). Enriched cells were injected subcutaneously into nude mice and a palpable tumor was observed five months later. The authors carried out a longitudinal study and samples were collected at two different time points (metastasis and progression) during the course of the disease, which allowed real-time assessment of molecular changes between patient tumor, CTCs, and CDXs samples. The obtained CDX phenocopied the patient tumor. Most importantly, RNA sequencing of the CDX tumor disclosed key mechanisms relevant in TNBC biology such as the WNT pathway, which is necessary for the maintenance of CSCs and was shown to correlate with metastasis and poor clinical outcome in TNBC subtypes. CTC analysis also deciphered a panel of potential tumor biomarkers [65]. An additional TNBC CDX model of liver metastasis was established very recently by Vishnoi et al. Similar genomic profiling of metastatic tissue was obtained in four sequential CDX generations, representing the recapitulation of liver metastasis in all the models. Notably, the authors deciphered a first 597-gene CTC signature related to liver metastasis in TNBC which, despite small sample size bias, can provide insight into the mechanistic basis of TNBC disease progression in the liver [66].

In melanoma, Girotti et al. demonstrated the tumorigenicity of advanced-disease CTCs in immunocompromised mice. The authors resorted to CDX development when tumor material was inaccessible for PDX generation. They reported a success rate of 13% with six CDX established, 15 failed attempts, and 26 additional models followed at the time of publication. CDX tumor growth was detectable as of one month after CTC implantation and was sustainable in secondary hosts. Moreover, the CDXs were representative of patient tumors and mirrored therapy response. This proof-of-principle was developed along with PDX technology and circulating tumor DNA analysis as part of a platform to optimize precision medicine for melanoma patients. It explored the TIC properties of melanoma CTCs but did not achieve a biological characterization of these cells [67].

In lung cancer, Hodgkinson et al. showed that CTCs in chemosensitive or chemorefractory SCLC are tumorigenic. CTCs were isolated from six late-stage SCLC patients having never received chemotherapy and were subsequently injected into NSG mice. Each patient presented with more than 400 CTCs and four out of six CTC samples gave rise to CDX tumors detected as of 2.4 months post-implantation. CDXs recapitulated the genomic profile of CellSearch-enriched CTCs and mimicked donor patients response to standard of care chemotherapy (platinum and etoposide), proving the clinical relevance of these models [68]. CDX tumors were subsequently dissociated and expanded into short-term in vitro CDX cultures (Table 2). These cells maintained the genomic landscape of donor tumors as well as their drug sensitivity profiles. CDX-derived cells were also labeled in vitro with the GFP lentivirus and successfully implanted into mice, where they can serve as a tracking tool to study tumor dissemination patterns in vivo [69]. Additional 16 SCLC CDX models were recently generated by Drapkin et al. from CTCs collected at initial diagnosis or at progression, with 38% efficiency. Somatic mutations were maintained between patient tumors and CDX as shown by whole-exome sequencing (WES) and the genomic landscape remained stable throughout early CDX passages showing clonal homogeneity. The authors also developed serial CDX models from one patient at baseline of the combination olaparib and temozolomide and at relapse. Interestingly, the models accurately reflected the evolving drug sensitivity profiles of the patient’s malignancy, which highlights the potential utility of serial CDXs to study the evolution of resistance to treatment in SCLC [70]. One CDX model was also described in NSCLC. In this study, CTC samples were retrieved at two different time points: Baseline and post-brain radiotherapy. No CDX was developed at baseline. Notably, no EpCAM^+^ CTCs were detected during CellSearch analysis at disease progression, yet injection of post-radiotherapy CTCs gave rise to a palpable tumor 95 days after engraft. Phenotypic and molecular characterizations showed no epithelial CTCs, but revealed a sizeable population of phenotypically heterogeneous CTCs mostly expressing the mesenchymal marker vimentin. This study suggests that the absence of EpCAM^+^ CTCs in NSCLC does not preclude the existence of CTCs with TIC potential in patients and underlines the importance of investigating CTCs undergoing EMT in this malignancy [71].

Our group generated the first CDX model of castration-resistant prostate cancer (CRPC) and derived a permanent ex vivo culture from CDX tumor cells. A total of 22 samples from metastatic CRPC patients were collected, among which seven were obtained from diagnostic leukapheresis (DLA). DLA products were generated as part of the European FP7 program CTCTrap which aimed for an increased CTC yield to perform molecular characterization of the tumor [72]. One patient with a very high CTC count (~20,000 CTCs) obtained by DLA gave rise to a palpable tumor within 5.5 months. Acquisition of key genetic drivers (i.e., *TP53, PTEN*, and *RB1*) that govern the trans-differentiation of CRPC into CRPC-neuroendocrine (CRPC-NE) malignancy was detected in CTCs, highlighting the role of tumorigenic CRPC-NE CTCs in this transformation. Moreover, the obtained in vitro CDX-derived cell line faithfully recapitulated the genetic characteristics and tumorigenicity of the CDX and mimicked patient response to standard of care treatments for CRPC (i.e., enzalutamide and docetaxel) (Table 2) [73,74].

## 5. CTC-Derived Ex Vivo Models

Although CDXs represent classical preclinical mouse models that are relatively easy to handle, they cannot be derived from every patient depending on tumor type and the process could take several months, a time frame that would not provide proper aid for the clinical guidance of donor patients. Expansion of viable CTCs ex vivo may offer an attractive alternative allowing both molecular analysis and high-throughput drug screening in a shorter time, but with CTC scarcity remaining, a fortiori, a significant limitation. In vitro CTC cultures were reported in colon, breast, prostate, and lung cancer and are evaluated in this section (Table 3).

The first long-term colon cancer CTC cell line was derived by Cayrefourcq et al. from a metastatic colon cancer patient who had 302 EpCAM^+^ CTCs detected by the CellSearch platform. Importantly, the characterized CTC-MCC-41 cell line shared the main genomic features of both the donor patient primary tumor and lymph node metastasis [9]. In a second study, the authors established and characterized eight additional cell lines from the same patient with CTCs collected at different time points during his follow-up. Transcriptomics analyses in the nine cell lines revealed an intermediate epithelial/mesenchymal phenotype promoting their metastatic potential, as well as stem cell-like properties that increased in cell lines isolated at later stages of progression. This may highlight the selection mechanism of treatment-resistant clones with specific phenotypes that drive disease progression. Functional experiments showed that these cells favor angiogenesis in vitro, which was concordant with the secretion of potent angiogenesis inducers such as VEGF and FGF2 as well as the tumorigenicity of these cells in vivo [9,10].

In BC, Zhang et al. presented the characterization of EpCAM^−^ CTCs and revealed a shared protein signature HER2^+^/EGFR^+^/HPSE^+^/Notch1^+^ in CTCs competent for brain metastasis. Indeed, the three established CTC lines expressing this signature promoted brain and lung localization after xenotransplantation into nude mice. The authors therefore deciphered a preliminary signature which provides insight into metastatic competency of BC CTCs and pushes towards using CTC research to explore new potential biomarkers [75]. Another study reported the establishment of non-adherent CTC lines under hypoxic conditions (4% O_2_) with CTCs issued from six patients with metastatic luminal-subtype BC. Three out of five tested cell lines were tumorigenic in vivo, giving rise to tumors with histological and immunohistochemical similarities with the primary patient tumor. This proof-of-concept study also identified targetable mutations acquired de novo in CTC cell lines, elucidating the importance of monitoring the mutational evolution of the tumor throughout the disease. To explore this, the authors performed sensitivity assays on the CTC lines with large panels of single drug and drug combinations targeting the different mutations identified [76]. In vitro phenotypic analysis of these cell lines and patient CTCs was recently performed. A CTM-specific DNA methylation status was revealed in which binding sites for stemness and proliferation transcription factors were hypomethylated, suggesting potential targets. This pattern correlated with poor prognosis in patients and targeting of clusters with Na+/K+ ATPase inhibitors shed them into single cell and enabled DNA methylation remodeling, leading to suppression of metastasis. These data therefore highlight a key connection between phenotypic properties of CTCs and DNA methylation patterns at specific stemness- and proliferation-related sites [43].

Despite successful in vitro expansion of patient CTCs in several cancer types as reported above, important limitations should be noted when handling 2D cultures, including cell morphology alterations due to adherence to plastic and lack of tumor microenvironment. Moreover, cell-cell and spatial interactions in vitro are not fully representative of the setting in the tumor mass in vivo [79]. These constraints can thus interfere with physiological functions and molecular responses of the tumor cells, making them less representative of the actual malignancy. To circumvent this problem, 3D models were proposed in prostate and lung cancer [77,78]. In prostate cancer, Gao et al. generated the first seven fully characterized organoid lines from a CRPC patient including a CTC-derived 3D organoid system from a patient who had more than 100 CTCs in 8 mL of blood. Success rate for the establishment of the CTC-derived organoid was not provided. Whole-exome sequencing (WES) analysis showed that all the 3D models recapitulated the molecular diversity of prostate cancer subtypes and were amenable to pharmacological assays. Engraftment of the CTC-derived organoid in vivo gave rise to tumors with a histological pattern similar to that of the primary cancer. This research, therefore, contributes a patient-derived model of CRPC which, with further optimization, may respond to the pressing need of in vitro models that faithfully recapitulate CRPC [77]. In lung cancer, Zhang et al. developed a novel ex vivo CTC-derived model using a 3D co-culture system which stimulated a microenvironment to sustain tumor development. CTCs were enriched and expanded for a short period of time from 14 to 19 early lung cancer patients. Next-generation sequencing detected several mutations including *TP53* found in both cultured CTCs and matched patient primary tumors [78].

## 6. Discussion

**Figure 1:**
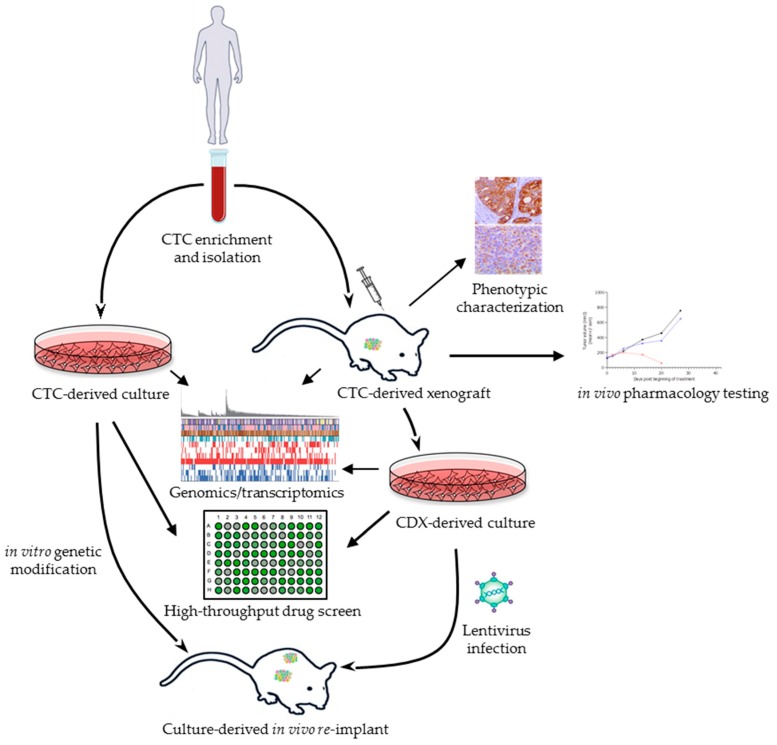
CTC-derived models as tractable systems to explore tumor-initiating cells (TICs) and new therapeutic strategies. CTCs isolated from late-stage cancer patients are used to generate CTC-derived xenografts (CDXs) to perform functional characterizations and pharmacology studies. CDX tumors can be isolated and dissociated into ex vivo cultures for drug screening and genome-wide analyses. CDX-derived cultures are amenable to lentiviral infection and can be re-injected into mice and used as tools to track tumor dissemination. In parallel, CTCs can be expanded in vitro and used as readouts of drug sensitivity. CTC = circulating tumor cell. CDX = CTC-derived xenograft.

During the last decade, tremendous technological progress has been made to reliably detect, quantify and characterize CTCs at phenotypic, genomic, and functional levels. The characterization of CTC-derived models has paved the way toward an improved understanding of tumor dissemination by these cells (Figure 1). As depicted in Table 1, procedures for developing CDXs can vary from one study to another. Subcutaneous (SC) injection of cells in mice is the simplest method for tumor engrafts which has been used for decades and was most recently applied for PDX establishment. It facilitates tumor growth monitoring as it does not require fluorescent labeling or imaging. Most CDX models published to date have been developed through SC injection of CTCs. SC tumors do not usually metastasize probably due to the absence of the human microenvironment and the impact of murine angiogenesis, which influence dissemination of primary human tumors. Moreover, as the time-frame needed for tumor growth extends to several months, ethical regulations may not allow waiting for metastatic spread. To this end, these studies were limited to the characterization of the CDX primary tumor. Injection in mouse bone marrow as done by Baccelli et al. may also be an appropriate way to investigate MICs as this microenvironment has been previously described as a reservoir for disseminating tumor cells [35,59]. Conversely, studies aiming to assess metastatic and not only tumorigenic competency of CTCs have resorted to intracardiac injection [66,75]. This method, similarly to tail vein (TV) injection, allows a more rapid spread of the cells as they directly enter the bloodstream and thus mimics CTCs in their original setting. Propagation of CDX models through intracardiac or TV injection is less common or completely lacking, most likely due to potential dissemination bias. Indeed, organ metastasis could be influenced by the injection site of CTCs and defined by the first capillary bed encountered by cells post-injection. TV has been observed to induce lung metastases, thus generating false-positive results [80].

Another important challenge is ensuring the CDX consistently maintains its clinical relevance and serves as a patient surrogate. To this end, stringent validation is required and several aspects must be addressed. Firstly, it is crucial to verify the human origin of the CDX, as spontaneous tumors could grow in immunocompromised mouse models. Secondly, confirming cancer type and comparing the CDX tumor to the donor patient’s biopsy through histopathology, followed by genomic studies to assess CDX genomic fidelity with patient tumor. Moreover, in the context of establishing preclinical models for precision medicine, functional drug sensitivity assays are needed to evaluate recapitulation of patient response to therapy in the CDX [68].

Although PDX models serve as reliable tools for tumor modeling, CDXs offer added value for the understanding of tumor biology and metastasis. Detection and characterization of metastasis-competent CTCs using in vivo models offer a more representative molecular snapshot of the disease, as they serve as easily accessible “surrogates” of metastatic tissue, which is otherwise unobtainable in many cancer organs (e.g., bones or lungs) [81]. Indeed, CDX models could help showcase tumor heterogeneity in the metastatic setting in contrast to a localized biopsy in the case of PDX and are attainable at different time points throughout disease progression [63]. Most importantly, CDX models established to date reveal the high tumorigenic capacity of CTCs—even at a low number of cells (as low as 400 CTCs [68]). As reviewed above, CTCs with survival and MIC properties are assumed to be selected for seeding CDX tumors, similar to what has been observed in PDXs [82]. It is expected that the proportion of tumorigenic CTCs may vary between cancer types and patients as well as under selective pressure of treatment, which highlights a potential selective process for the acquisition of minor metastasis-competent CTC subclones [73]. CTC clusters and hybrid E/M CTCs have been described as the most aggressive cells with a high propensity for tumorigenesis. However, it is currently difficult to evaluate the impact each subpopulation could have on CDX tumor take rate. Indeed, as detailed before, Aceto et al. have shown an increased metastatic competency in CTM vs. single CTCs but this remains limited to murine models and is difficult to translate to human subjects [40]. It is worth noting however that, although in vivo models are sustained by the host tissue microenvironment and can faithfully recapitulate the tumor genome, the absence of immune components constitutes an important bias.

On the other hand, CTC expansion ex vivo is promising but is still very far from routine applications as culturing conditions are still under investigation and need further optimization. Therefore, CDX-derived cultures represent an attractive intermediate model to characterize this aggressive population in vitro. In the event of molecular similarities between the two models, CDX and CDX-derived cell lines offer complementary, tractable systems for CTC functional characterization and therapy testing. Re-injection of the CDX-derived cell line in immunodeficient mice could allow the identification of candidate genes in metastasis and chemoresistance mechanisms [64,69]. Additional model systems such as the chick embryo chorioallantoic membrane have also opened up new promising avenues in the in vivo studies of tumor metastasis, as the highly vascularized setting sustains tumor formation and dissemination rapidly after engraft [83]. Moreover, organoids have recently emerged as novel robust 3D models optimized to propagate in vitro and reminiscent of tumoral heterogeneity, with amenability to genetic modifications and drug screening assays [77,84]. One can hypothesize that the establishment of several CTC-derived organoid lines from the same patient could be useful in modeling metastatic disease and acquired CTC mutational profiles to monitor disease progression. However, these models lack in vivo host complexity and recent efforts have been put into the generation of 3D co-cultures in microfluidic devices to model ex vivo tumor microenvironments by the integration of different cell populations (e.g., immune cells, fibroblasts) [78,85].

## 7. Concluding Remarks

CDX models have shown unprecedented opportunities to provide insight into the complex biology of the metastatic process. However, at the present time, these functional models serve as proof-of-principle tools as their development is limited to late-stage disease settings and high CTC counts. The main goal of functional CTC studies being the identification and characterization of MICs and candidate target genes among CTCs, it is crucial to expand analyses to earlier stages of cancer [63]. Unfortunately, these rare preclinical models are derived from patients in exceptional clinical situations and we are currently unable to predict if the limitation caused by CTC scarcity could be circumvented. Nevertheless, the establishment of CTC-derived models from only a few CTCs is a major achievement today and an invaluable opportunity to decipher new biomarkers, which are urgently needed for novel therapeutic strategies in advanced cancers.

## Figures and Tables

**Table 1:** Overview of in vivo circulating tumor cell (CTC)-derived models established to date.

*CTC-Derived Xenografts*								
Type of Cancer	Stage	Live CTC Isolation Technique	# of CTCs	Injection Procedure	Take Rate	Passaging	Main Findings	Ref
**Breast cancer**	Metastatic luminal	FACS isolation (PI^−^CD45^−^EpCAM^+^) or RosetteSep	≥1109 CTCs EpCAM^+^(CellSearch)	- Dilution in matrigel - Injection in femoral medullar cavity	5%	N/A	- Specific CTC MIC signature EpCAM^+^CD44^+^MET^+^CD47^+^ - Recapitulation of patient metastases phenotype in CDX metastases - No drug sensitivity study	[59]
	Metastatic triple-negative	Density gradient centrifugation: Histopaque^®^	969 CTCs EpCAM^+^ (CellSearch)	- Dilution in matrigel - Subcutaneous injection	3%	Piece of tumor explant or injection of explant culture	- RT-qPCR for genomic profiling of CTC/CDX samples before and after injection - WNT pathway upregulation as a potential therapeutic target in TNBC identified by RNAseq - No drug sensitivity study	[65]
	Metastatic triple-negative	FACS (CD45^−^/CD34^−^/CD105^−^/CD90^−^ CD73^−^)	N/A	Intracardiac injection	33%	Minced metastatic liver tissue	- Identification of a TNBC liver metastasis CTC-specific signature (whole-transcriptome)- Survival analyses for signature transcripts	[66]
**Melanoma**	Stage IV	RosetteSep	N/A	- Dilution in matrigel - Subcutaneous injection	13%	Tumor fragments	- recapitulation of patient response to dabrafetinib in the CDX - concordance in SNV profiles (WES/RNAseq)	[67]
**SCLC**	Metastatic	RosetteSep	>400 CTCs EpCAM^+^ (CellSearch)	Dilution in matrigel/subcutaneous	67%	Tumor fragments	- Recapitulation of CTC genomic profile by CDX tumors - CDX mimicked donor’s response to chemotherapy	[68]
	Limited or extensive stage	CTC-iChip + RosetteSep Ficoll	N/A	Dilution in matrigel/subcutaneous	38%	Tumor fragments	- Faithful recapitulation of the tumor genome - Reflection of evolving treatment sensitivities of patient tumor	[70]
**NSCLC**	Metastatic	RosetteSep	>150 CTCs by FACS (CD45/CD144/ vimentin/CK)	Dilution in matrigel/subcutaneous	100%	Disaggregation of tumor	- Importance of mesenchymal CTCs with tumorigenic capacity	[71]
**CRPC**	Metastatic	DLA/RosetteSep	~20,000 CTCs EpCAM^+^ (CellSearch)	Dilution in matrigel/subcutaneous	14%	Tumor fragments	- Recapitulation of genome characteristics in CTC, patient tumor and CDX (WES) - Tumorigenic CTCs with acquired CRPC-NE features	[73]

* N/A: not available; FACS: Fluorescent-activated cell sorting; CDX: CTC-derived xenograft; MIC: Metastasis-initiating cell; TIC: Tumor-initiating cell; TNBC: Triple-negative breast cancer; SCLC: Small-cell lung cancer; SNV: Single nucleotide variant; NSCLC: Non-small cell lung cancer; CRPC: Castration-resistant prostate cancer; NE: Neuroendocrine; WES: Whole-exome sequencing.

**Table 2:** Overview of CDX-derived ex vivo cultures established to date.

*CDX-Derived* Ex Vivo *Cultures*				
Type of Cancer	Stage	Culturing Conditions	Main Findings	Ref
**SCLC**	Metastatic	HITES medium with ROCK inhibitor—non-adherent cell clusters—short-term	Recapitulate genomic landscape and in vivo drug response Tumorigenic in vivo Lentiviral transduction of one cell line	[69]
**CRPC**	Metastatic	DMEM/F12 medium—adherent conditions—permanent	Recapitulation of genomic characteristics and standard of care drug response	[73]

**Table 3:** Overview of ex vivo CTC-derived models established to date.

*CTC-Derived* Ex Vivo *Models*							
Type of Cancer	Stage	Live CTC Isolation Technique	# of CTCs (CellSearch)	Culturing Conditions	Success Rate	Main Findings	Ref
**Colon cancer**	Nonresectable metastatic	RosetteSep	≥300	- Hypoxic in medium 1 DMEM/F12 to normoxic conditions in medium 2 RPMI1640 - 2D, sustained for >6months	1%	- Recapitulation of main genomic features - Tumorigenic in vivo - Intermediate EMT + stem cell properties	[9,10]
**Breast cancer**	Metastatic	FACS	0	- Normoxic stem cell culture medium - 2D	8%	- Tumorigenic in vivo, brain metastasis signature (EpCAM^−^HER2^+^/EGFR^+^/HPSE^+^/Notch1^+^)	[75]
	Metastatic luminal	CTC-iChip	3–3000	- Hypoxic, nonadherent - 2D, Sustained for >6 months	83%	- Tumorigenic in vivo - Drug sensitivity panels and CTM-specific methylation profile	[43,76]
**CRPC**	Metastatic	RosetteSep-Ficoll	>100	- Growth factors reduced Matrigel/Advanced DMEM/F12 - 3D, sustained for >6 months	6%	- Tumorigenic in vivo	[77]
**NSCLC**	Early stage	Microfluidic CTC-capture device	1–11	- Matrigel + collagen - 3D, sustained for ~1 month	73%	- Common mutations between cultured CTCs and primary tumor	[78]
